# Supplementary material for: Complexome profiling of the Chlamydomonas psb28 mutant reveals TEF5 as an early PSII assembly factor
Source: Plant Cell. 2025 Mar 17;37(6):koaf055. doi: 10.1093/plcell/koaf055 (PMC12164590; doi:10.1093/plcell/koaf055)
Supplement: koaf055_Supplementary_Data [file koaf055_supplementary_data.zip › psb28_interactive.zip!/plots/intensity/qualityPlots/peptides/FAP12_Cre09.g390615.t1.1.html]

### Cre09.g390615.t1.1

Trivial name: FAP12  
SeqCoverage [median]: 28.80  
Mass: 52.14 kDa  
  
 

| Sequence | MissedCleavage | Mass [Da] | wtIntensity | muIntensity | Score |
| --- | --- | --- | --- | --- | --- |
| AALVVSMAAEPTPK | 0 | 1383.74 | 0.00 | 312.89 | 69.98 |
| DGGDGNTASAPQPK | 0 | 1313.58 | 0.00 | 681.27 | 178.49 |
| GTANMKDVLTDLAGAAR | 1 | 1702.87 | 0.00 | 5005.99 | 97.84 |
| IIKEPEYAK | 1 | 1089.61 | 946.85 | 9036.25 | 91.87 |
| IYGGVPMPGKK | 1 | 1145.63 | 0.00 | 526.26 | 44.32 |
| KEAAEGESFGSDLK | 1 | 1466.69 | 0.00 | 1481.94 | 58.07 |
| LGQPAVAVVADRER | 1 | 1479.82 | 0.00 | 1415.01 | 42.32 |
| QVELQSLQDTGYHVDAK | 0 | 1929.94 | 0.00 | 684.27 | 58.67 |
| SLPDHLAATEDPTNQVEK | 0 | 1963.95 | 0.00 | 2942.96 | 51.70 |
| TLKVEDVAYFR | 1 | 1339.71 | 0.00 | 1809.57 | 78.34 |
|  |  |  | 946.9 | 23896.4 |  |
